# Supplementary material for: The chemotherapeutic drug CX-5461 is a potent mutagen in cultured human cells
Source: Nat Genet. 2023 Nov 30;56(1):23–6. doi: 10.1038/s41588-023-01602-9 (PMC10786719; doi:10.1038/s41588-023-01602-9)
Supplement: Supplementary file 1 — Reporting Summary [file 41588_2023_1602_MOESM1_ESM.pdf]

nature portfolio

Corresponding author(s): Prof Serena Nik-Zainal

Last updated by author(s): Oct 22, 2023

Reporting Summary

Nature Portfolio wishes to improve the reproducibility of the work that we publish. This form provides structure for consistency and transparency in reporting. For further information on Nature Portfolio policies, see our Editorial Policies and the Editorial Policy Checklist.

Statistics

For all statistical analyses, confirm that the following items are present in the figure legend, table legend, main text, or Methods section.

n/a	Confirmed	
☐	☒	The exact sample size (n) for each experimental group/condition, given as a discrete number and unit of measurement
☐	☒	A statement on whether measurements were taken from distinct samples or whether the same sample was measured repeatedly
☐	☒	The statistical test(s) used AND whether they are one- or two-sided<br>*Only common tests should be described solely by name; describe more complex techniques in the Methods section.*
☒	☐	A description of all covariates tested
☒	☐	A description of any assumptions or corrections, such as tests of normality and adjustment for multiple comparisons
☐	☒	A full description of the statistical parameters including central tendency (e.g. means) or other basic estimates (e.g. regression coefficient) AND variation (e.g. standard deviation) or associated estimates of uncertainty (e.g. confidence intervals)
☐	☒	For null hypothesis testing, the test statistic (e.g. F, t, r) with confidence intervals, effect sizes, degrees of freedom and P value noted<br>*Give P values as exact values whenever suitable.*
☒	☐	For Bayesian analysis, information on the choice of priors and Markov chain Monte Carlo settings
☒	☐	For hierarchical and complex designs, identification of the appropriate level for tests and full reporting of outcomes
☒	☐	Estimates of effect sizes (e.g. Cohen's d, Pearson's r), indicating how they were calculated

Our web collection on statistics for biologists contains articles on many of the points above.

Software and code

Policy information about availability of computer code

Data collection	We performed whole genome sequencing and TwinStrand duplex sequencing of all experimental control and treatment samples on Illumina Novaseq 6000 platform generating 150 base pair paired-end reads.<br><br>Mutagen exposure WGS data of human induced pluripotent stem cells (used for calculating mutagenicity index) were published and were accessed via https://data.mendeley.com/datasets/m7r4msjb4c/2.
Data analysis	Experimental WGS short read data were aligned to the human reference genome GRCh38 assembly using “bwa mem 0.7.17-r1188”. Quality control and bioinformatic analysis of the WGS data was performed using “CaVEMan v1.13.15 “ for substitutions, “Pindel v3.2.0” for insertions/deletions, “Brass v6.2.1” for rearrangements, and “ASCAT (NGS) v4.2.1” for copy number variations. Experimental signature derivation was performed as described in doi: 10.1038/s43018-021-00200-0 and codes can be obtained from https://github.com/xqzou/COMSIG_KO and https://rdrr.io/github/Nik-Zainal-Group/signature.tools.lib/.<br><br>TwinStrand error-corrected duplex sequencing data analysis was performed using DuplexSeq Mutagenesis App v4.1.0 hosted on DNAnexus using default parameters, Pipeline ID: human-muta-v1.0.<br><br>Drug sensitivity profiling data were analysed with GraphPad version 9.5.1.

For manuscripts utilizing custom algorithms or software that are central to the research but not yet described in published literature, software must be made available to editors and reviewers. We strongly encourage code deposition in a community repository (e.g. GitHub). See the Nature Portfolio guidelines for submitting code & software for further information.

Data

Policy information about availability of data

All manuscripts must include a data availability statement. This statement should provide the following information, where applicable:

- Accession codes, unique identifiers, or web links for publicly available datasets
- A description of any restrictions on data availability
- For clinical datasets or third party data, please ensure that the statement adheres to our policy

Raw sequence files from hTERT-RPE1 and HAP1 mutation accumulation experiments are deposited at the European Genome-Phenome Archive with accession number EGAD50000000036. Mutation calls have been deposited at Mendeley and can be accessed via DOI: 10.17632/d58cv549v6.1. Downstream analyses data are provided in the Supplementary Tables. All cell line models cells can be requested directly from the corresponding author. Curated data are available for general browsing from Signal (https://signal.mutationalsignatures.com) upon publication.

Research involving human participants, their data, or biological material

Policy information about studies with human participants or human data. See also policy information about sex, gender (identity/presentation), and sexual orientation and race, ethnicity and racism.

Reporting on sex and gender	N/A
Reporting on race, ethnicity, or other socially relevant groupings	N/A
Population characteristics	N/A
Recruitment	N/A
Ethics oversight	N/A

Note that full information on the approval of the study protocol must also be provided in the manuscript.

Field-specific reporting

Please select the one below that is the best fit for your research. If you are not sure, read the appropriate sections before making your selection.

☒ Life sciences ☐ Behavioural & social sciences ☐ Ecological, evolutionary & environmental sciences

For a reference copy of the document with all sections, see nature.com/documents/nr-reporting-summary-flat.pdf

Life sciences study design

All studies must disclose on these points even when the disclosure is negative.

Sample size	From a statistical standpoint, this was an exploratory study, and there were no pre-defined hypothesis tests for which sample-size power calculations were appropriate.
Data exclusions	From a statistical perspective, this was an exploratory study, and there were no pre-defined hypothesis tests for which pre-defined data exclusion criteria would have been appropriate. Therefore, no data were excluded from by our algorithms.
Replication	Each gene edit/treatment arm had at least 2-4 daughter sub-clones as biological replicates. For TwinStrand duplex sequencing experiment, each treatment arm only has one duplex library being sequenced and analysed. Drug sensitivity profiling data were obtained from a minimum of three independent replicates. All attempts at replication were successful.<br><br>For few of the chemicals (for example, CX-5461), we replicated the analysis to identify CX-5461 associated signatures using different cell line models. For all the chemicals, each of the subclone genomes sequenced represents an independent data point and as such much of the paper explored the replication of signatures across the cell line collection.
Randomization	The question of allocation to experimental groups is not applicable to this study. No randomization was performed. All experimental samples were contrasted against respective isogenic unedited (WT) or untreated (DMSO solvent treated) controls.
Blinding	We applied the analysis algorithms to each and every perturbation (i.e., treatment) in the dataset in exactly the same way and without any prior expectations about the desired outcome of the analysis. Therefore, blinding was not required.

Reporting for specific materials, systems and methods

We require information from authors about some types of materials, experimental systems and methods used in many studies. Here, indicate whether each material, system or method listed is relevant to your study. If you are not sure if a list item applies to your research, read the appropriate section before selecting a response.

Materials & experimental systems

n/a	Involved in the study
☒	☐ Antibodies
☐	☒ Eukaryotic cell lines
☒	☐ Palaeontology and archaeology
☒	☐ Animals and other organisms
☒	☐ Clinical data
☒	☐ Dual use research of concern
☒	☐ Plants

Methods

n/a	Involved in the study
☒	☐ ChIP-seq
☒	☐ Flow cytometry
☒	☐ MRI-based neuroimaging

Eukaryotic cell lines

Policy information about cell lines and Sex and Gender in Research

Cell line source(s)	The original hTERT RPE-1 are hTERT-immortalized retinal pigment epithelial cells derived by transfecting the RPE-340 cell line with the pGRN145 hTERT-expressing plasmid. This is a near-diploid human cell line of female origin with a modal chromosome number of 46 that occurred in 90% of the cells counted. All hTERT-RPE1 cells that we used in this study was originally generated from doi: 10.1038/s41586-018-0291-z. They were gifts from M. Tarsounas, (Department of Oncology, University of Oxford, UK). HAP1 cell was obtained from J. Loizou (CeMM, Austria, DOI: 10.1038/s41467-017-01439-x). Human induced pluripotent stem cells were initially described in DOI: 10.1016/j.cell.2019.03.001. The human induced pluripotent stem cell (hiPSC) was derived at the Wellcome Trust Sanger Institute (Hinxton, UK). The use of this cell line model was approved by Proportionate Review Sub-committee of the National Research Ethics (NRES) Committee North West - Liverpool Central under the project "Exploring the biological processes underlying mutational signatures identified in induced pluripotent stem cell lines (iPSCs) that have been genetically modified or exposed to mutagens" (ref: 14.NW.0129).
Authentication	The cell lines were not authenticated in this study. However, we did have the whole genome-sequencing data and had matched SNP genotype profiles to confirm the cell line identities and their isogenicity.
Mycoplasma contamination	Stock cell lines were tested negative for mycoplasma contamination when banked and used the first time, but not tested again throughout the mutation accumulation, drug exposure, and single-cell subcloning steps.
Commonly misidentified lines (See ICLAC register)	Not applicable as none were used.
